# Temperature-Responsive Pyraclostrobin-Loaded Octadecane Submicrocapsules with Lowered Toxicity

**DOI:** 10.3390/nano10122374

**Published:** 2020-11-28

**Authors:** Aiym Tleuova, VSSL Prasad Talluri, Rohitesh Kumar, František Štěpánek

**Affiliations:** 1 Department of Chemical Engineering, University of Chemistry and Technology Prague, Technická 5, 166 28 Prague 6, Czech Republic; Frantisek.Stepanek@vscht.cz; 2 Department of Biotechnology, University of Chemistry and Technology Prague, Technická 5, 166 28 Prague 6, Czech Republic; 3 Department of Physical Chemistry, University of Chemistry and Technology Prague, Technická 5, 166 28 Prague 6, Czech Republic; 4 Department of Biochemistry and Microbiology, University of Chemistry and Technology Prague, Technická 5, 166 28 Prague 6, Czech Republic; rohku@dtu.dk

**Keywords:** fungicide, pyraclostrobin, octadecane, phase-change material, microencapsulation, Pickering emulsion, triggered release

## Abstract

Pyraclostrobin (Pyr) is one of the most effective fungicides. However, it can degrade via photolysis in water, it is toxic to aquatic life and if inhaled, it has a low solubility in water, that leads to difficulties when applying to plants by spraying. Additionally, the necessity of repeated (weekly) sprays of fungicides when the pathogen growth risk is the highest, such as at the temperature range of 24 to 36 °C and increased humidity of about 95%, leads to loss of efficiency of the fungicide and overdose of chemicals. In the present study, pyraclostrobin was microencapsulated to solve the abovementioned issues. As a core of capsules octadecane (OD) with a melting point of 28 °C was used, thus, the release of pyraclostrobin was controlled via temperature change. Pyraclostrobin-loaded submicrocapsules (PyrSMCs) were characterized using SEM, DLS, TGA/DSC, HPLC, FTIR methods; stimuli-responsivity was tested employing in vitro tests with pathogenic culture (Fungal strain of *Pyrenophora teres* - CPPF-453) grown in Petri dishes. Toxicity of PyrSMCs to *Artemia salina* was studied as well. Size of capsules was 200–600 nm along with the presence of bigger capsules with a diameter of 1–4 µm. PyrSMCs showed excellent antifungal effects above the melting point of octadecane. PyrSMCs demonstrated 29 times less toxicity than pyraclostrobin of technical grade. Overall, results show the potential of such capsules to be applied in the agricultural industry for precise agriculture strategies.

## 1. Introduction

The importance of agriculture has always been and remains one of the most important productions for humans, especially, in the 21st century, when population has increased and reached its maximum. However, plant diseases can cause significant pre-harvest crop loses. Fungi are one of the most dangerous challenges to food security [1], especially because they can easily be spread through air and water.

Plant protection products (PPPs) used to be the main tool to control plant diseases, even though they are known to be toxic [2] and need a forecasting system to be applied on time when the risk of pathogen growth is the highest, such as in warm temperatures and moist environments.

One of the most effective fungicides, pyraclostrobin (methyl N-{2-[1-(4-chlorophenyl)-1H-pyrazol-3-yloxymethyl] phenyl}(N-methoxy) carbamate), is used to control the main fungal diseases that affect a whole range of crops, including apples. Pyraclostrobin is one of the most effective fungicides. Its importance in agriculture is not in doubt. It can control the main fungal diseases that affect many agronomic and fruit crops, such as cotton, wheat, corn, banana and others. However, when applying directly on the plant, pyraclostrobin can degrade via photolysis in water with a half-life (t_1/2_) of 1.7 days, thus, reducing its efficacy. It is known to be toxic to aquatic life and if inhaled, it has low solubility in water that leads to difficulties when applying on the plants by spraying.

One of the ways to solve the abovementioned problems can be encapsulation as a delivery system in plants [3,4,5,6]. Encapsulation can help to avoid its degradation and reduce a toxic effect on the environment; however, while designing delivery system, it is essential to study its toxicity [7].

Pyraclostrobin encapsulation systems were reported in recent works. L. Cao and others [8] obtained N-(2-hydroxyl)propyl-3-trimethyl ammonium CS chloride (HTCC) capped pyraclostrobin-loaded mesoporous silica nanoparticles (MSNs) with an initial burst and subsequent sustained release behavior. Pyraclostrobin-loaded HTCC-capped MSNs with half doses of pyraclostrobin of technical grade showed almost the same fungicidal activity against *Phomopsis asparagi* (Sacc.), thus, reducing the amount of applied pesticide and enhancing its efficiency.

Li and co-workers [9] developed pyraclostrobin-loaded microcapsules (MCs) using coordination assembly between Fe^3+^ and tannic acid (TA) to promote the efficacy and environmental safety of pyraclostrobin. After eight Fe^3+^-TA deposition cycles, nearly all the MCs retained a stable spherical shape with an average diameter of 9 μm. MCs resulted in significantly higher yields at doses of 120 and 180 g ha^−1^ and showed lowered toxicity to *Brachydanio rerio*, *Daphnia magna*, *Xenopus laevis*, and *Rana nigromaculata.*

Pyraclostrobin-loaded polyurea microcapsules with a size distribution from 0.815 to 8.674 μm, and encapsulation efficiency of more than 90% were reported in [10]. The release was controlled by diffusion and erosion. Microcapsule formulations showed significantly decreased toxicity to zebrafish, high spreadability on rice leaves, and satisfactory control efficacy against rice panicle blast (85.03%).

Pyraclostrobin-loaded poly (lactic-co-glycolic acid) (PLGA) nanospheres with a diameter of 0.6 µm, an active ingredient loading of 17.2%, and a loading rate of 89.7% showed enhanced ultraviolet resistance [11]. Authors also reported a faster release rate in slightly acidic and slightly basic environments compared to the release in a neutral condition, as well as that smaller particles had a faster release rate.

Fungicides are usually sprayed several times per season when the pathogens growth risk is the highest, such as in the temperature range of 24 to 36 °C and increased humidity of about 95% [12].

Wang et al. [13] reported temperature-responsive hybrid capsules with a phase-transition temperature of 27 °C. Capsules with a size of 5–10 µm were synthesized through water-in-oil Pickering emulsion polymerization. The emulsion was stabilized by silica-modified nanoparticles and the aqueous phase consisted of N-isopropyl acrylamide and poly (N-isopropyl acrylamide); the size of capsules was about 5–10 µm. Authors compared the release rate of a model drug (Rhodamin B) at 25 °C and 45 °C within 35 h, which amounted to 25.7% and 70.9%, respectively.

A reusable temperature-responsive core-shell structure with particle diameters of 1–2 mm was developed for fertilizer delivery by co-authors [14]. Ethylene oxide/propylene oxide block copolymer (F-127) played the main role in controlling Fe^2+^ release via opening and closing pores at different temperatures. The liquid state (at the temperature range of 25–35 °C) covers a small part of the pores, thus, promoting the release, and beyond temperatures of 25–35 °C, it closes pores through the liquid–gel transition.

When designing temperature-responsive capsules (TRCs) based on a phase-change material (PCM) core, the shell properties are an important factor affecting thermal characteristics, such as thermal stability and conductivity [15]. Silica particles are widely used for Pickering emulsions preparation due to their nontoxicity, high surface area and ability to provide mechanical stability to the formulation; particularly, this allows the prevention of the leakage of melted oil phase in TRCs [16].

Microcapsules shells consisting of silica particles provide higher thermal conductivity for the core of capsules and enhance thermal energy storage and release abilities. He and co-workers [17] demonstrated that n-octadecane microcapsules with silica shell provide increased thermal stability and thermal conductivity compared to those of pure n-octadecane.

Kim et al. [18] prepared octadecane microcapsules with a waterborne polyurethane shell and found that increasing octadecane content leads to an increase in the heat of fusion and crystallization of encapsulated octadecane.

Considering the abovementioned evidence, temperature-responsive capsules of pyraclostrobin can contribute in the preventive protection of plants and in reducing the necessity of repeated (weekly) sprays of fungicides, thereby reducing the toxicity of fungicides. Thus, when the temperature is below PCM melting point, the release rate must be minimal, but when the temperature starts to grow due to liquid state of the core material, the diffusion of pyraclostrobin increases and it releases out to the surface, thus, protecting the plant from the fungi. Therefore, the aim of the present work is to provide a temperature-responsive property to pyraclostrobin fungicide using 3-(Trimethoxysilyl)propyl methacrylate (TPM)/SiO_2_ core/shell submicrocapsules (SMCs) containing PCM n-octadecane in oil phase and study the toxicity of synthesized submicrocapsules.

## 2. Materials and Methods

### 2.1. Materials

Technical grade pyraclostrobin, ≥95% (SAGECHEM, Hangzhou, China) was used as a fungicidal active ingredient. Octadecane, ≥99% was used as phase-change material of oil phase and was purchased from Merck in Darmstadt, Germany. 3-(trimethoxysilyl)propyl methacrylate (TPM) with the purity of 98% (Merck, Germany) was used as a matrix of the oil phase. Bindzil 50/80 (Eka Chemicals, Gothenburg, Sweden), wt.50% silica dioxide nanoparticles with pH10 and size of up to 80 nm were used as a shell material of submicrocapsules. Potassium persulfate K_2_S_2_O_8_ (KPS) (SigmaAldrich, Darmstadt, Germany) was used as an initiator of the polymerization process. The fungal strain of *Pyrenophora teres*—CPPF-453 was provided by the Crop Research Institute (Prague, Czech Republic). *A.salina* cysts (Sera artemia mix) were purchased from Invital aqua s.r.o, Opava, Czech Republic. Chemicals and reagents were used as received without further processing.

### 2.2. Preparation of Submicrocapsules

**Figure 1:**
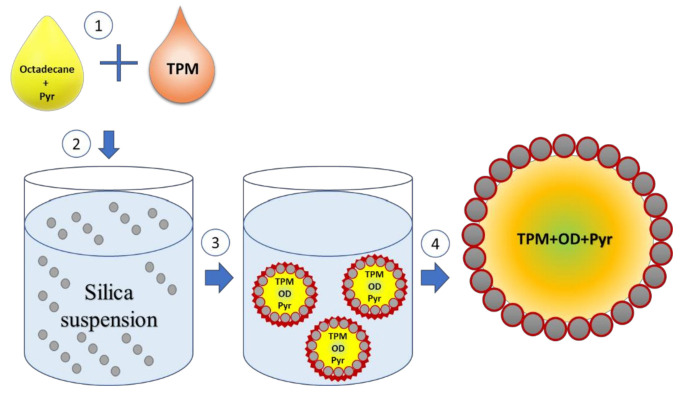
Illustration of the procedure of submicrocapsules preparation.

The schematic illustration of the synthesis of submicrocapsules is shown in Figure 1. The melting point of octadecane is about 28.2 °C, therefore, it was heated up to 35 °C before use. The temperature was maintained throughout the synthesis process using a heater nest. Pyraclostrobin was added, the resulting mixture was heated up to 45 °C and stirred at 300 rpm for 30 min. Then, a certain amount of TPM was added dropwise and the mixture was further stirred at 300 rpm for 30 min. The resulting mixture appearance was transparent with a light yellow color. Meanwhile, the silica nanoparticles (SiNPs) suspension was diluted with water and heated up to 45 °C. The oil mixture was added into the aqueous phase (silica nanoparticles suspension), then the water was added up to the mark of 50 mL. To obtain an emulsion, ultrasonication (Sonopuls, Bandelin, Germany) was applied at 30% power in pulsing mode (2.5 s of pulsation with 0.5 s of pauses) for 5 min. The resulting Pickering emulsion was cooled down to room temperature and was polymerized for 1 h at 80 °C using KPS with a concentration of 0.4 mM. The concentration of the oil phase in the aqueous phase of emulsion remained constant (4.3% of the oil phase). The weight ratio of silica particles to the oil phase was 0.2. The OD:TPM ratio in the oil phase studies for antifungal activity was 1:4 and 2:3. Pyraclostrobin concentration in the oil phase varied between 1 and 10%.

### 2.3. Characterization of Submicrocapsules

Dynamic light scattering (DLS): The size of SMCs was measured using the Malvern ZetaSizer Nano-ZS apparatus (Kassel, Germany).

Scanning electron microscopy (SEM): The SMCs surface was studied by means of scanning electron microscopy (JCM-5700, JEOL, Tokyo, Japan). The samples were gold-plated before SEM imaging.

Fourier-transform infrared (FTIR) analysis: FTIR measurements were carried out using NICOLET 6700 spectrometer (Thermo Scientific, Waltham, MA, USA) with Omnic 7.0 software. Samples were prepared using potassium bromide pellet and recorded over the spectral region of 4000–400 cm^−1^.

High performance liquid chromatography (HPLC): The load content (LC) was determined by HPLC (1200-DAD, Agilent, Santa Clara, CA, USA). Here, 2.0 mg of pyraclostrobin-loaded SMCs was dissolved in 10.0 mL of methanol under stirring for 48 h at 35 °C. Then the SMCs were centrifuged at 10,000 rpm for 10 min and filtered using filters with a 0.2 µm pore size. The LC was calculated according to formula: LC (%) = (weight of fungicide encapsulated in SMCs/weight of SMCs) × 100. The HPLC operating parameters were as follows: Venusil XBP-C18 column (Bonna-Agela Technologies Inc., Tianjin, China) (2.5 mm × 4.6 mm, 5 µm thickness); column temperature: 30 °C; mobile phase: methanol; flow rate: 1.0 mL/min; injection volume: 10 µm; diode array detector (DAD) signals: 275 nm.

Thermogravimetric analysis (TGA): The thermal property of the SMCs was examined using simultaneous thermogravimetry-differential scanning calorimetry (TG-DSC) (Setaram Sensys Evo thermal analyzer, Caluire, France). TG-DSC was equipped with a symmetrical balance and a Calvet 3D sensor. The temperature ranges from 15 to 800 °C were inspected with a heating rate of 10 °C/min. Nitrogen flow was 20 mL/min.

### 2.4. Temperature-Responsivity of SMCs via Antifungal Activity

Stimuli-responsivity of SMCs at different values of temperature was tested employing in vitro tests with pathogenic culture (Fungal strain of *Pyrenophora teres*—CPPF-453) grown in Petri dishes. Fungal strain *Pyrenophora teres* was received from Agricultural Crop Research Institute (Prague, Czech Republic) and was used for antifungal activity assay. The fungal strain was maintained on sterile PD agar (Potato Dextrose agar) medium at 4 °C for further use.

For antifungal activity, *P. teres* was aseptically inoculated on sterile PDA petri dishes (autoclaved, cooled and settled PDA medium) and spread uniformly [19]. Wells of approximately 4 mm in diameter and 2.5 mm deep were made on the surface of solid agar medium using a sterile borer.

Then, 100 µL of each suspension of blank SMCs and loaded with fungicide was transferred into the wells. Pyraclostrobin of technical grade was used as a positive control at a concentration of 50, 250, 500 μg/mL. The tests were carried out in triplicates in two sets. One set of plates at 25 °C and another set of plates was incubated at 35 °C for 7 days to investigate the temperature-responsive release of pyraclostrobin from the capsules. After the incubation period, the growth was observed. Since the *P. teres* is highly sporulating fungi and tend to spread throughout the plate, it has been found to be complicated to measure the inhibition zone, and the deviation of the collected data was high. Therefore, in this work it was decided to carry out the visual estimation of fungi growth as a proof of concept, which allowed the comparison of the growth of fungi at two different temperatures.

### 2.5. Toxicity of PyrSMCs Using Brine Shrimps (Artemia salina)

#### 2.5.1. Culture and Harvesting of A. Salina

A packet of 18 g containing artificial sea salts and *A. salina* cysts were incubated for hatching in a 1 L glass beaker filled with 500 mL of distilled water. Cysts were incubated at 27 °C temperature under white-fluorescent light and maintained the aeration with the help of air sparging. After 24 h, hatched nauplii (larva) were attracted by light which allowed their aggregation at one place. The active vital nauplii were collected by using pipette from the light side and transferred into fresh artificial seawater for further use.

#### 2.5.2. Preparation of Sample

First, the stock solutions of blank SMCs, pyraclostrobin-loaded submicrocapsules (PyrSMCs) with initial concentration of 0.516 mg/mL, were prepared. Then, test solutions at appropriate concentrations in the range of 50–2500 µg/mL were prepared from stock solution by diluting with water. Control tests were carried out by using artificial seawater without the presence of any SMCs. All tests were conducted in triplicates.

#### 2.5.3. Toxicity Test

Toxicity test were conducted according to the Harwig and Scott method [20]. An amount of 10 mL of filtered artificial seawater solution was added into petri dishes (70 mm × 15 mm). Then, 10 active nauplii were transferred into each petri dish using a micropipette. Certain amounts of SMCs were added into the artificial seawater and were maintained under illumination at 25 °C temperature. After 24 h and 48 h, surviving nauplii were counted with the help of a 5X magnifying glass and the mortality rates (percentage of deaths) at the different doses and the control one were determined.

In cases where control deaths occur, the data were corrected using Abbott’s formula as follows: % deaths = (Control-Test)/Control × 100.

Here, test means number of survived nauplii. The mean percentage of lethality was plotted against the concentrations and the concentration killing 50% of the nauplii was determined from the graph. The median lethal concentrations (LC50) for the tested SMCs samples were calculated using the slope with at least an 85% confidence interval and 5 concentration points for calculation of each equation. LC50 was calculated by using the mortality rate for 24 and 48 h.

## 3. Results and Discussion

### 3.1. Capsules Characterization

Alkoxysilane 3-(trimethoxysilyl)propyl methacrylate (TPM) tends to emulsify spontaneously in the presence of solid nanoparticles, such as silica and magnetite [21]. Such Pickering emulsion forms due to surface modification of hydrophilic solid nanoparticles by TPM hydrolysis product 3-(Trihydroxysilyl)propyl methacrylate which are known to be surface active [22]. Further polymerization of emulsion droplets allows producing SMCs loaded with active ingredients, such as lubricants [23] and biocides [24]. Thus, TPM/silica SMCs can be used as a platform for encapsulation of different hydrophobic ingredients, including fungicides.

As it is known, the silica nanoparticles amount influences size and polydispersity of final capsules [25]. It was previously reported that at the ratio m(SiO_2_)/V(TPM) of 0.43, the average size of SMCs is 210 nm [23]. Decreasing the amount of silica nanoparticles leads to the stabilization of less surface area and, therefore, the formation of bigger droplets.

The addition of octadecane into TPM affects the interfacial properties of oil phase and requires a greater external force to break the interface. It is assumed that this will lead to the formation of bigger droplets than those with pure TPM. Kim et al. [18] reported that the size of octadecane microspheres increased with increasing octadecane contents, while the size of microspheres decreased with increasing emulsifier content. Wang and co-workers [13] reported that size of capsules depend on number of silica nanoparticles. Considering this, it was decided to choose the lower m(SiO_2_)/m(oil) ratio of 0.2, which will be enough to stabilize bigger droplets with less surface area.

**Figure 2:**
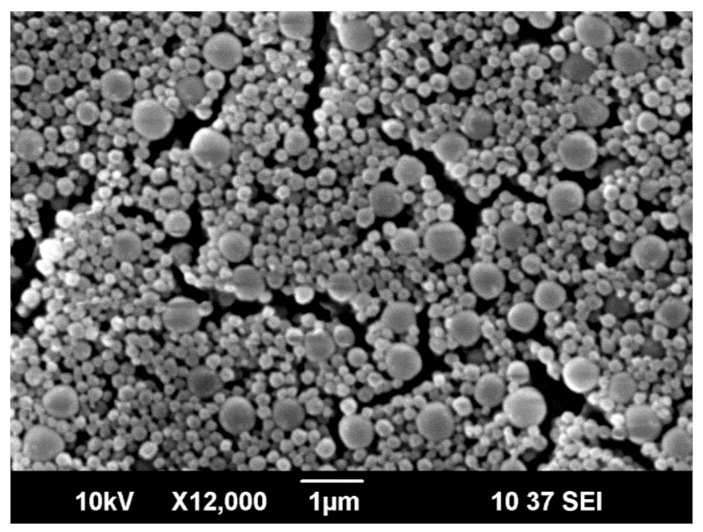
SEM image of 3-(Trihydroxysilyl)propyl methacrylate (TPM) submicrocapsules (SMCs) when silica/TPM weight ratio was 0.2.

SEM image of the SMCs of TPM/SiO_2_ with the ratio m(SiO_2_)/m(TPM) of 0.2 is shown in the Figure 2. DLS measurement showed SMCs with size of 200–600 nm. This result was used as a starting point before addition of octadecane and pyraclostrobin in the encapsulation system. The effect of the addition of octadecane and pyraclostrobin at different ratios on the morphology of SMCs was studied further.

The addition of octadecane in the oil phase did not allow us to emulsify the system spontaneously, therefore ultrasonication was utilized to emulsify the mixture at the temperature above its melting point. Octadecane concentration in the oil phase was varied from 10 to 90%.

After addition of pyraclostrobin in the oil phase in amount of 1%, 5% and 10%, the concentration of the other two components were reduced, however, their weight ratio remained. For example, for OD 10% and TPM 90%, the ratio became 1:9; OD 20%—OD:TPM 1:4, OD 20%—3:7, OD 40%—2:3, OD 50%—1:1, OD 60%—3:2, OD 90%—9:1. Therefore, the content of OD and TPM in oil phase will be shown in ratios.

The melting point of pyraclostrobin is about 63.7–65.2 °C. When preparing oil phase before emulsification, a certain amount of pyraclostrobin was added into octadecane at 35 °C. The resulting mixture was heated up to 45 °C and stirred at 300 rpm for 30 min. However, small particles of pyraclostrobin were still visible and to fully dissolve them it was necessary to heat the mixture up to melting point of pyraclostrobin. Otherwise, when TPM was added dropwise in the mixture of OD and pyraclostrobin at 45 °C, the solution became transparent with light yellow color. This means pyraclostrobin is soluble in TPM even at temperatures of 35–45 °C, allowing us to carry out the emulsification at 40–45 °C.

Introduction of the higher amount of octadecane in the oil phase leads to complication of emulsification and visible loss of encapsulation efficiency. The layer of oil phase can be observed when the ratio of OD amount in OD:TPM ratio is higher than 2:3. Additionally, the resulting emulsions showed less stability compared to emulsions with lower content of OD.

**Figure 3:**
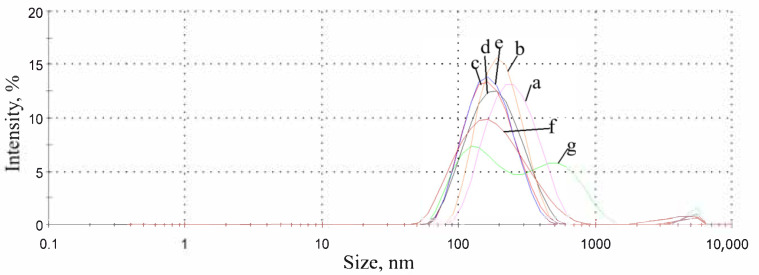
Size distribution by intensity of pyraclostrobin-loaded submicrocapsules (PyrSMCs) depending on octadecane (OD):TPM weight ratio in the oil phase. a—1:9; b—1:4; c—3:7; d—2:3; e—1:1; f—3:2; g—9:1. Silica/oil phase—weight ratio of 0.2.

The change of morphology can be noticed from size distribution measurements results. In Figure 3, size distribution by intensity shows increasing polydispersity and the appearance of additional peaks, such as at the ratio of OD:TPM 9:1. DLS showed the average size of PyrSMCs about 220 nm with the presence of bigger capsules with the size of 1–4 µm.

**Figure 4:**
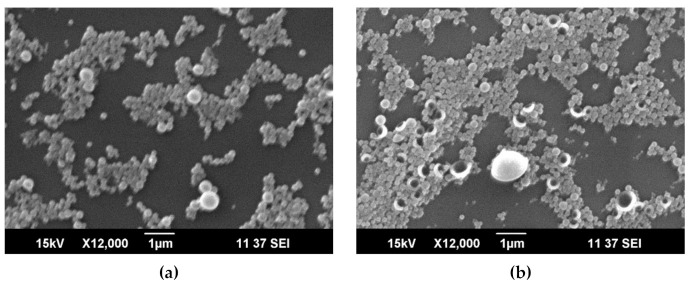

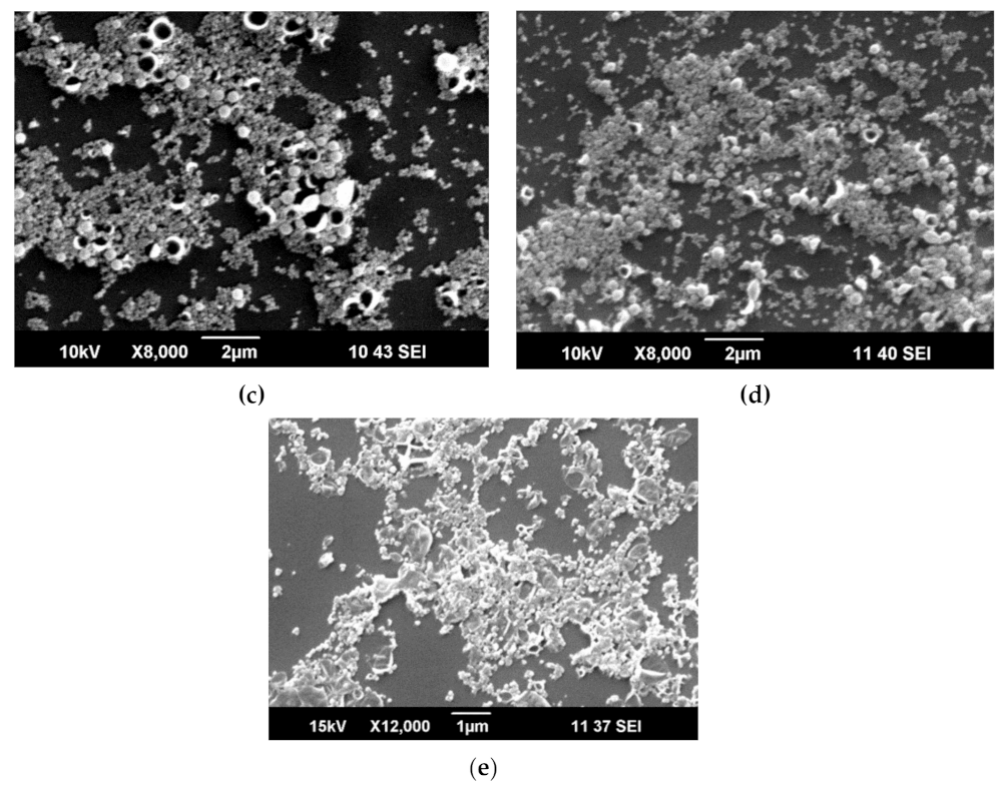
SEM images of pyraclostrobin-loaded submicrocapsules depending on OD:TPM ratio in the oil phase. (**a**)—1:9; (**b**)—1:4; (**c**)—3:7; (**d**)—2:3; (**e**)—3:2. The concentration of pyraclostrobin (Pyr) in oil phase of 1%.

The SEM images of PyrSMCs at various concentration of octadecane in the oil phase are shown in Figure 4. The appearance of higher number of bigger capsules takes place with the increase in OD in oil phase. The OD:TPM ratio of 2:3 was found to be critical (Figure 4d); the further increase in the OD in oil phase led to low stability of the emulsion (Figure 4e).

Considering the morphology results of the submicrocapsules, the systems with an OD:TPM ratio of 1:4 and 2:3 were chosen for further studies.

At this stage, the effect of pyraclostrobin concentration in oil phase on morphology, composition, antifungal activity and toxicity of SMCs, was studied. Pyraclostrobin content in the oil phase was varied from 1 to 10%. Its weight concentration was calculated by the oil phase. The total mass of oil phase remained constant and was equal to 2.15 g. For example, for the sample containing 10% of pyraclostrobin (Pyr) in oil phase, the total mass of oil phase consisting of OD, TPM and Pyr was 2.15 g, where mass of Pyr was 0.215 g. The ratio of OD:TPM remained the same, as in samples without Pyr. The composition of the emulsion system is shown in Table 1. Aqueous phase was added up to the mark of 50 mL.

**Figure 5:**
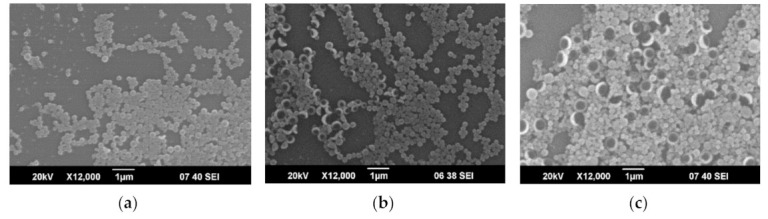
SEM images of PyrSMCs depending on pyraclostrobin concentration in oil phase. (**a**)—1%; (**b**)—5%; (**c**)—10%.

**Figure 6:**
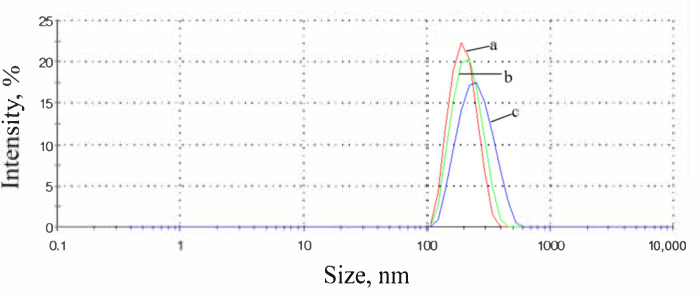
Size distribution by intensity of PyrSMCs depending on pyraclostrobin content in the oil phase: a—1%; b—5%; c—10%. Silica/oil phase—weight ratio of 0.2.

SEM images of PyrSMCs demonstrated round-shaped spheres (Figure 5). With an increase in Pyr content in oil phase of SMCs, an increase in the amount of the bigger particles can be observed. This can be noticed from the DLS results in Figure 6 that show a shift in spectra of size towards bigger sizes (1%—191 nm; 5%—206 nm; 10%—238 nm) and higher polydispersity (1%—0.05; 5%—0.06; 10%—0.09). Zeta-potential of obtained PyrSMCs were also changed with change of Pyr concentration: for Pyr 1%—52.5 mV; for Pyr 5%—47.7 mV and for Pyr 10%—45.7 mV.

The resulting SMCs content after washing and drying was calculated considering the presence of silica NPs. The calculated composition of dried SMCs is shown in Table 2. Here, Pyr concentration was calculated by the total mass of SMCs including SiNPs. This was important to study the load content. For example, for SMCs containing 10% of Pyr in oil phase, the Pyr content in SMCs was 8.3%.

**Figure 7:**
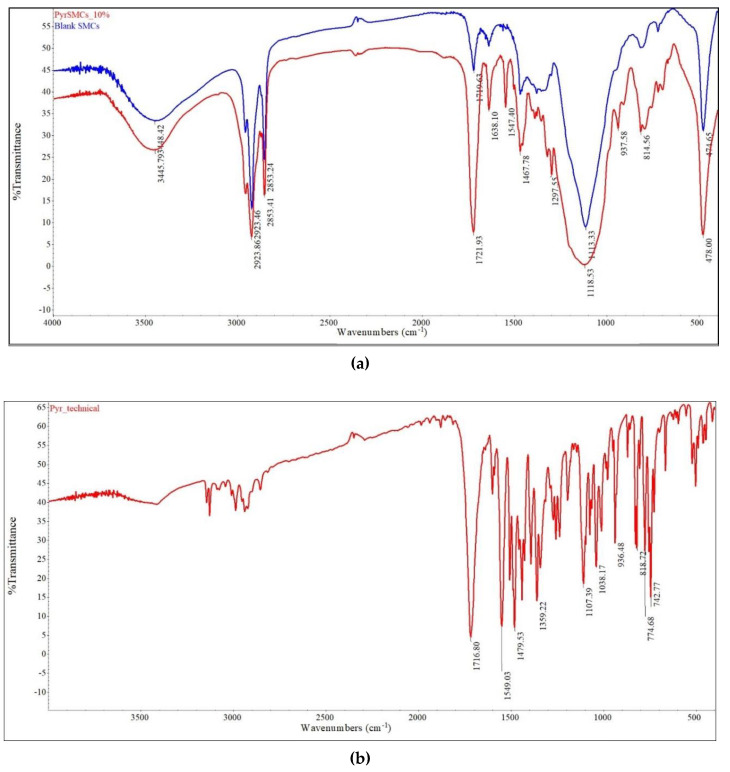
Infrared spectrogram: (**a**)—SMCs (red–blank SMCs, blue–PyrSMCs), (**b**)—pyraclostrobin of technical grade.

To confirm the composition of PyrSMCs, the FTIR spectroscopy of pyraclostrobin of technical grade, blank SMCs and PyrSMCs was carried out and is shown in Figure 7.

PyrSMCs show an intense absorption at 1721.93 cm^−1^ reflecting the stretching vibration of the C = O aliphatic ketones that present in the structure of both pyraclostrobin and TPM molecules. The peak at 1547.40 cm^−1^ was associated with the stretching vibration of the benzene ring skeleton. The peak at 937.58 cm^−1^ corresponded to the adsorption of C-N stretching vibration.

The characteristic band absorptions of pyraclostrobin and blank SMCs that were detected in PyrSMCs demonstrated the successful encapsulation of pyraclostrobin in TPM/OD SMCs.

HPLC analysis showed that the loading content of SMCs is higher than that calculated and shown in Table 2. For SMCs with pyraclostrobin concentration in oil phase of 1% (0.83% in SMCs), the loading content in SMCs was 0.91%. The loading rate of pyraclostrobin in SMCs is shown in Table 3.

It is suggested that the increase in the concentration of pyraclostrobin in SMCs is due to hydrolysis of TPM during emulsification and its partial diffusion into aqueous phase. This leads to the change of the remaining concentration in oil phase, therefore leading to the increase in the load content of pyraclostrobin in SMCs. In addition, it can be noticed that with the decrease in TPM initial concentration in oil phase, the load rate of pyraclostrobin decreases as well. This means that less TPM hydrolyzes and passes into aqueous phase and the composition of the SMCs remains similar to the initial one. The amount of hydrolyzed TPM during emulsification and its influence on final load content of SMCs must be studied in more detail in further research.

It is also assumed that some amount of pyraclostrobin might pass into the aqueous phase. However, the HPLC analysis of aqueous phase of emulsion did not show the presence of pyraclostrobin, which can be explained by the adsorption of hydrophobic molecules of pyraclostrobin on the partly hydrophobic surface of SMCs.

**Figure 8:**
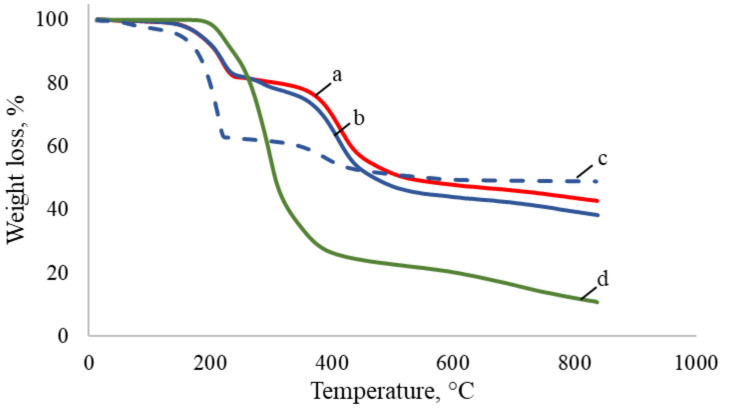
TGA curves: a—PyrSMC with 10% Pyr and OD:TPM 1:4; b—blank SMCs with OD:TPM 1:4; c—blank SMCs with OD:TPM 2:3; d—pyraclostrobin of technical grade.

The TGA spectrograms of PyrSMCs, blank SMCs and pyraclostrobin of technical grade are illustrated in Figure 8.

For pyraclostrobin of technical grade (Figure 8, curve d), the rapid weight loss started at about 200 °C up to550 °C and consisted of a single stage due to its decomposition. The residual amount of pyraclostrobin of technical grade at 800 °C was 12.1%.

There are two degradation stages that were observed in TGA curves for all SMCs (Figure 8, curves a, b, c). The first stage belongs to OD decomposition in both blank (Figure 8, curve b, c) and 10% Pyr-loaded SMCs (Figure 8, curve a) which takes place from 150 to 240 °C.

TPM weight loss can clearly be seen starting from about 260 °C when two curves of blank (Figure 8, curve b) and Pyr-loaded SMCs (Figure 8, curve a) start to differ. This can be explained by the TPM slow degradation in the temperature range of 260–360 °C. Due to the higher amount of TPM in blank SMCs, the weight loss in blank SMCs is more compared to PyrSMCs.

At the third stage, TPM weight loss was also observed in the period of 350–500 °C in blank and Pyr-loaded SMCs.

For a blank SMCs sample with OD:TPM ratio of 2:3 (Figure 8, curve c), the first degradation stage is more rapid and the weight loss is higher; this proves the higher amount of octadecane in the capsules. At the same time, the second degradation stage is lower, that belongs to TPM decomposition for which the concentration is lower.

Thus, TGA findings confirm the successful encapsulation of pyraclostrobin in SMCs.

**Figure 9:**
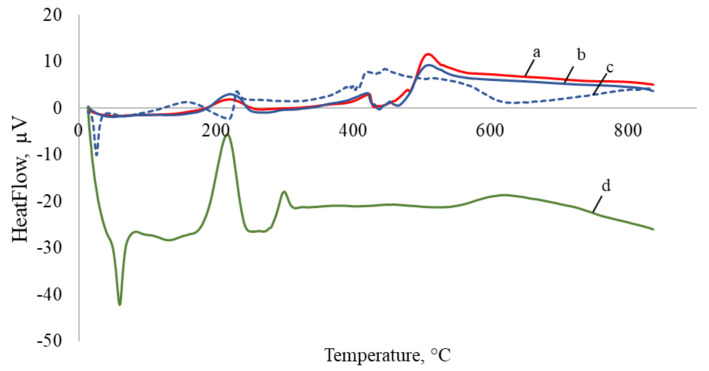
DSC curves: a—PyrSMC with 10% Pyr and OD:TPM 1:4; b—blank SMCs with OD:TPM 1:4; c—blank SMCs with OD:TPM 2:3; d—pyraclostrobin of technical grade.

The DSC spectrogram of PyrSMCs, blank SMCs and pyraclostrobin of technical grade are illustrated in Figure 9.

DSC results showed a sharp endothermic peak at 25 °C for blank SMCs with OD:TPM ratio of 2:3 (Figure 9, curve c) compared to SMCs with OD:TPM ratio of 1:4 (Figure 9, curves a, b). This demonstrates the better thermal activity of SMCs with OD:TPM ratio of 2:3 due to higher concentration of OD in oil phase, which is in line with the literature. Thus, Kim et al. [18] reported that increase in the amount of n-octadecane leads to increase in the heat of fusion and crystallization.

For pyraclostrobin of technical grade, the first endothermic peak at 65 °C belongs to the melting point of pyraclostrobin; the second exothermic peaks at 216 °C and 299 °C relate to the degradation of pyraclostrobin. These peaks are almost not visible in PyrSMCs, which demonstrates that pyraclostrobin was coated by the microcapsule shell.

### 3.2. Temperature-Responsive Properties through Fungicidal Activity

*Pyrenophora teres* is a necrotrophic fungi which is pathogenic to plants; in particular, it causes net blotch on barley (*Hordeum vulgare*) worldwide. This fungal strain was used for antifungal activity (AFA) tests to study thermal responsivity of SMCs. For this purpose, SMCs with different OD:TPM ratios and pyraclostrobin concentrations were taken. Table 4 demonstrates the antifungal activity of SMCs with OD:TPM ratio of 2:3.

The SMCs with OD:TPM ratio of 2:3 loaded with 1%, 5% and 10% pyraclostrobin incubated at 25 °C did not show any anti-fungal activity but showed luxuriant growth of *P. teres.* (Table 4). No antifungal effect can be observed because below the melting point of octadecane, the pyraclostrobin almost does not diffuse from the oil phase. The blank SMCs do not show any zone of inhibition at both 25 and 35 °C, that clearly indicates silica nanoparticles; OD and TPM do not exhibit any effect on the *P.teres.* SMCs loaded with 5% and 10% pyraclostrobin exhibited a good antifungal activity and have the potential to inhibit the growth of fungi *P.teres* incubated at 35 °C. This is due to the release profile of the pyraclostrobin from the SMCs at 35 °C. The SMCs with OD:TPM ratio of 1:4 showed similar results. The visual assessment of antifungal activity evidently shows that the SMCs work on basis of thermal response release. Thus, microencapsulation of Pyr in PCM allowed us to obtain temperature-triggered SMCs to control the fungal growth of *P. teres*.

### 3.3. Toxicity of PyrSMC

Pyraclostrobin was previously tested for toxicity using *Hyalella azteca* amphipods [26] and *B. cognatus* tadpoles [27] with LC50 values of 22.0 µg/L and 10.0 µg/L, respectively. Thus, this indicates the high toxicity of this fungicide. In this research, we used *Artemia salina* brine shrimps because of the ease of cultivation and conducting the tests. The positive control of technical grade pyraclostrobin shows the highest value in this test with the LC50 value of 64 µg/L at 24 h.

**Figure 10:**
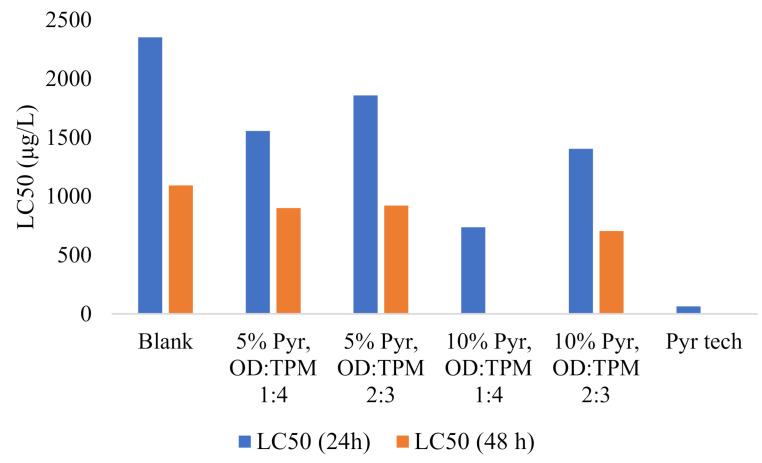
Toxicity of PyrSMCs with various compositions to *Artemia salina.*

Table 5 demonstrates the toxicity data of SMCs towards *Artemia salina* nauplii at 24 h and 48 h incubation time, as well as the regression equations and regression coefficient R^2^ values. The regression equations represent the dose–response of lethality. The regression analysis results indicated that mortality is directly correlated to the concentration of SMCs in water. LC50 of blank SMCs was 2352 µg/L at 24 h and 1092 µg/L at 48 h that clearly indicates their low toxicity towards brine shrimps. The SMCs showed twice more toxicity at 48 h compared to 24 h. This noticeably demonstrates that SMCs was toxic in terms of more incubation time. The PyrSMCs in comparison with pyraclostrobin of technical grade showed much less toxicity but they showed more compared to blank SMCs. This can be seen from Figure 10.

It was noticed that PyrSMCs toxicity depends directly on the ratio of concentration of pyraclostrobin. OD:TPM ratio also affects the LC50 values, as OD:TPM 1:4 is more toxic than OD:TPM 2:3. This can be explained by the state of OD in the core of SMCs. Toxicity testing was conducted at the temperatures below the melting point of OD, therefore, higher amount of OD entraps more Pyr and does not let it diffuse through the shell of SMCs.

Nevertheless, the overall PyrSMCs (5% Pyr, OD:TPM 2:3) are 29 times less toxic than pyraclostrobin of technical grade, which indicates significantly lowered toxicity to *Artemia salina*.

The anti-fungal tests showed that when the temperature rises above the melting point of OD, the diffusion of Pyr to the SMCs surface takes place. It is assumed that after temperature drops below the OD melting point, the diffusion of Pyr slows down and can be considered as negligible. It is expected that the remaining amount of Pyr on the surface of SMCs would increase the toxicity of SMCs to *Artemia salina.* Additionally, the prior temperature activation of Pyr formulations would lead to the release of some amount of pyraclostrobin to the surface of SMCs, which in turn would increase the toxicity of PyrSMCs. However, we assume that in the field works, at the same efficiencies of PyrSMCs and Pyr of technical grade, the amounts of Pyr necessary for the encapsulation is considerably less compared to conventional Pyr. Due to small amounts of Pyr, the toxicity of PyrSMCs would be still lower than of conventional Pyr. Therefore, the toxicity is reduced due to both encapsulation and the reduction in the amount of pyraclostrobin.

## 4. Conclusions

An OD/TPM/SiNPs system was successfully utilized to microencapsulate pyraclostrobin. The FTIR, TGA and HPLC confirmed the chemical composition of obtained SMCs. The morphology of SMCs depends on the amount of octadecane and pyraclostrobin. The SMCs showed spherical core-shell structure with the average size of 200–600 nm with the presence of bigger capsules with a diameter of 1–4 µm. DSC results showed better thermal activity of SMCs with OD:TPM ratio of 2:3 compared to SMCs with OD:TPM ratio of 1:4 due to a higher concentration of OD in oil phase. The antifungal activity results evidently showed that the SMCs work on the basis of thermal response release. PyrSMCs loaded with 5% and 10% pyraclostrobin exhibited a good antifungal activity against *P.teres* at 35 °C, while below the melting point of octadecane, the PyrSMCs did not demonstrate any antifungal activity due the lack of diffusion of pyraclostrobin. The PyrSMCs showed twice more toxicity at 48 h compared to that at 24 h. Toxicity testing demonstrated that microencapsulation of pyraclostrobin lowered its toxicity to *Artemia salina* by 29 times compared to pyraclostrobin of technical grade.

## Figures and Tables

**Table 1:** The composition of emulsion depending on pyraclostrobin content (OD:TPM ratio is 1:4), g.

Composition, g	Pyraclostrobin Concentration in the Oil Phase, %			
	-	1%	5%	10%
SiNPs	0.43	0.43	0.43	0.43
OD	0.43	0.4257	0.4085	0.387
TPM	1.72	1.7028	1.634	1.548
Pyr	-	0.0215	0.1075	0.215
Aqueous phase	50 mL	50 mL	50 mL	50 mL

**Table 2:** The composition of PyrSMCs depending on Pyr concentration in the oil phase, %.

Composition, %	Pyraclostrobin Concentration in the Oil Phase, %			
	-	1%	5%	10%
SiNPs	16.7	16.7	16.7	16.7
OD	16.7	16.5	15.8	15
TPM	66.7	66	63.3	60
Pyr	-	0.8	4.2	8.3

**Table 3:** The loading content of pyraclostrobin in SMCs depending on pyraclostrobin concentration and OD:TPM wt. ratio.

OD:TPM wt. Ratio	Pyraclostrobin Theoretical Concentration in SMCs, %		
	0.8%	4.2%	8.3%
0:1	0.96	5.065	11.088
1:4	0.91	4.43	9.54
2:3	0.908	3.708	8.561

**Table 4:** Antifungal activity for blank and Pyr-loaded SMCs at OD:TPM ratio of 2:3 at 25 and 35 °C.

OD:TPM 2:3	25 °C	35 °C
Blank	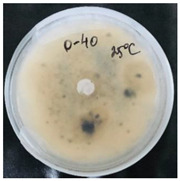	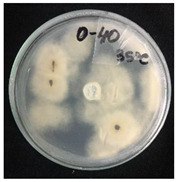
1% Pyr (41.28 µg)	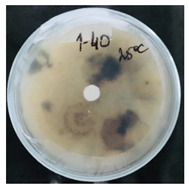	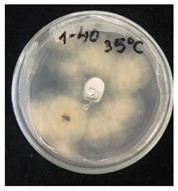
5% Pyr (216.72 µg)	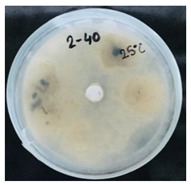	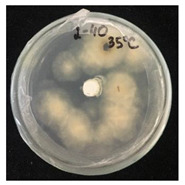
10% Pyr (428.28 µg)	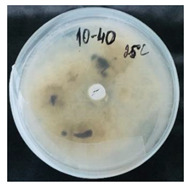	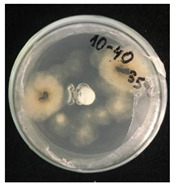
Pyr tech (500 µg)	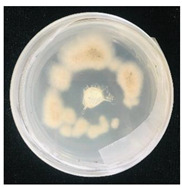	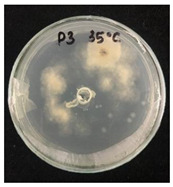

**Table 5:** Median lethal concentration (LC50) values of PyrSMCs with various compositions on *Artemia salina*.

Sample	LC50 (24 h)	Equation	R^2^	LC50 (48 h)	Equation	R^2^
Blank	2352	y = 0.0222x − 2.2222	0.89	1092	y = 0.0356x + 11.111	0.94
5% Pyr, OD:TPM 1:4	1556	y = 0.04x − 12.222	0.88	900	y = 0.0667x − 10	0.97
5% Pyr, OD:TPM 2:3	1858	y = 0.0311x − 7.7778	0.94	922	y = 0.0844x − 27.778	0.93
10% Pyr, OD:TPM 1:4	737	y = 0.0844x − 12.222	0.97	-	-	
10% Pyr, OD:TPM 2:3	1405	y = 0.0356x − 2 × 10^−14^	0.89	705	y = 0.0756x − 3.3333	0.94
Pyr tech	64	y = 0.2444x + 34.444	0.92	-	-	
